# Attainment of World Health Organization physical activity recommendations by Ecuadorian children: Analysis of social and anthropometrics factors in two distinct populations

**DOI:** 10.1371/journal.pone.0311165

**Published:** 2024-12-18

**Authors:** María José Molina-Cando, Irina Chis Ster, Samuel Escandón, René-Vinicio Sanchez, Alejandro Rodriguez, Martha E. Chico, Maritza Vaca, Christopher G. Owen, Delfien Van Dyck, Philip J. Cooper, Angelica Ochoa-Aviles

**Affiliations:** 1 Bioscience Department, Faculty of Chemistry, Universidad de Cuenca, Cuenca, Azuay, Ecuador; 2 Department of Movement and Sports Sciences, Faculty of Medicine and Health Sciences, Ghent University, Ghent, Belgium; 3 Institute of Infection and Immunity, St George’s University of London, London, United Kingdom; 4 Grupo de Investigación y Desarrollo en Tecnologías Industriales (GIDTEC), Universidad Politécnica Salesiana, Cuenca, Azuay, Ecuador; 5 School of Medicine, International University of Ecuador, Quito, Ecuador; 6 Independent Researcher, Quito, Ecuador; 7 Instituto de Saúde Coletiva, Universidade Federal do Bahia, Salvador, Brazil; 8 Population Health Research Institute, St George’s, University of London, London, United Kingdom; UBC: The University of British Columbia, CANADA

## Abstract

Daily adherence to WHO recommended physical activity guidelines has multiple health benefits in children and adolescents. Limited data from low and middle-income countries are available regarding adherence to WHO physical activity recommendations. This study aims to objectively measure physical activity intensities and explore associations with sociodemographic and anthropometric factors related to nonadherence to the WHO minimum physical activity recommendations. Two cross-sectional studies were conducted between 2014 and 2019 in two distinct populations of Ecuador in terms of poverty and residency (Cuenca: 66% live in urban areas, and 38.2% have unsatisfied basic needs; Quininde, 76.4% live in rural areas and 91% have unsatisfied basic needs). Waist-worn accelerometers were used to measure daily physical activity, standardized questionnaires were used to assess sociodemographic variables (age, sex, ethnicity, and socioeconomic status), and anthropometric (weight, height, waist circumference) measurements were taken. Multivariable regression was used to assess the relationship between sociodemographic and anthropometric factors with physical activity in each study population. The study involved 650 participants in Cuenca, with a mean age of 9.1 years (SD 2.9), and 985 children and adolescents in Quinindé, with a mean age of 8.3 years (SD 0.4). In both settings, boys were more likely than girls (Cuenca: adj. OR 3.09, 95% CI 2.17–4.39; Quinindé adj. OR 5.63, 95% CI 4.03–7.85) to achieve the WHO physical activity guidelines. More affluent participants were much less likely to meet this recommendation than their poorer counterparts in both settings. In Cuenca, a higher waist circumference was inversely associated with meeting WHO daily physical activity recommendation (adj. OR 0.96, 95% CI 0.94–0.98), whereas in Quinindé, adherence to WHO guidelines was more likely in non-Mestizo than Mestizo children (adj. OR 1.45, 95% CI 1.02–2.07). The findings suggest that anthropometric differences and sociodemographic disparities influence the attainment of WHO physical activity recommendations in Ecuadorian children.

## Introduction

Regular physical activity (PA) provides mental and physical health benefits [1]. An average of 60 minutes per day or more of moderate-to-vigorous PA (MVPA) in children and adolescents improves cardiorespiratory and muscular fitness, bone and cardiometabolic health, cognitive development, and prosocial behavior [2]. Despite these benefits, the majority of children and adolescents worldwide do not meet current PA guidelines [3, 4].

In low- and middle-income countries (LMICs), information on PA has been collected through subjective (self-report) questionnaires [5–7]. Although questionnaires are highly versatile, easy-to-administer, and cost-effective assessment tools, they are limited by recall bias and variation in reporting duration and accuracy for different intensities of PA, particularly in children [8, 9]. Objective PA assessment tools, such as accelerometers, can overcome these limitations. Accelerometers accurately estimate the intensity, volume, duration, and frequency of PA performed, hence adherence to PA guidelines. These parameters are useful for studies to determine relationships between health outcomes and risk factors [10].

Adherence to PA recommendations is generally lower among children and adolescents, in particular, girls tend to adhere less to the daily requirement of 60 minutes of MVPA compared to boys, and the prevalence of compliance with PA guidelines decreases with age [11]. Furthermore, children’s daily MVPA compliance has been described to differ significantly between and within countries. High daily compliance with the World Health Organization (WHO) PA guidelines has been observed in high-income countries except for the USA [12]. Moreover, rural children in low-income countries demonstrate higher compliance with the recommended 60 minutes of daily MVPA compared to their urban counterparts [13, 14].

In Ecuador, as in other LMICs, adherence to WHO PA recommendations has been mainly based on self-report [15, 16]. In fact, it has been observed that compliance with WHO guidelines of at least 60 minutes of MVPA daily is slightly higher when self-reported, in contrast to accelerometer-derived measurements [17]. Data on prevalence and correlates of compliance with the WHO PA guidelines in children and youth from LMICs, particularly using objective tools among representative samples of children, are limited [18]. Moreover, adherence to this recommendation at both the population level and within specific demographic groups, especially in rural settings, remains largely unknown [19, 20]. Such data, including information on socialdemographic and anthropometric characteristics affecting the achievement of these minimum recommended levels of PA, will be valuable to identifying disparities in access to PA opportunities and developing health promotion strategies for underprivileged groups [21]. In the present study, we used two samples of Ecuadorian children living in distinct settings to objectively measure time spent in sedentary behavior (SB) and PA at different intensities using accelerometers and explore sociodemographic and anthropometric factors associated with SB and PA at different intensities and non-adherence to WHO recommendations for PA.

## Methods

### Study settings and participant selection

Two cross-sectional studies were conducted between 2014 and 2019 in two distinct populations of Ecuador. The first study was conducted in Cuenca, Azuay Province, and the second in Quinindé, Esmeraldas Province. Cuenca, located in the Ecuadorian highlands at an altitude of 2550 m, is Ecuador’s third-largest city, with 505,585 inhabitants, 90% of whom are Mestizo. About 66% of the population lives in the city’s urban and peri-urban neighborhoods, and the remainder (34%) live in rural areas. Cuenca has an Unsatisfied Basic Needs (NBI, *Spanish acronym*) poverty indicator of 38.2%, in contrast to Quinindé canton, which has a significantly higher NBI of 91%. Quinindé is a District of Esmeraldas province, located in the northwest tropical region, with a 57–96% parish poverty incidence. The district serves a population of approximately 122.570 people with limited access to basic services, and it is largely rural (76% of the population) with economic activities based mainly on agriculture [22].

Detailed methodology of objectives, design, follow-up and sample selection for each study is provided elsewhere [23, 24]. In Cuenca, fifth to eighth-grade schoolchildren were invited to participate in the study between 25^th^ October 2018 and 24^th^ October 2019, encompassing the entire period of accelerometer data collection for this cohort. The study involved a sample of randomly selected schools of 1,028 children aged 9–12 from the urban area. Children without disabilities or conditions that might influence their regular PA (i.e., congenital disorders such as Down’s Syndrome, congenital heart defects, musculoskeletal disorders, cerebral palsy, as well as chronic diseases and hypothyroidism) and with a signed written consent were included [23]. The ECUAVIDA cohort was a population-based birth cohort of 2,404 children recruited at the time of birth in the public hospital (Hospital Padre Alberto Buffoni) in the town of Quinindé between 18^th^ November 2005 and 20^th^ December 2009, and the aims and methodology are described in detail elsewhere [24]. Follow-up of 1950 children still in the cohort was done at or after the child’s 8th birthday. For assessment of PA, cohort children were invited to participate when they reached eight years of age, with accelerometer data collection starting on 14th April 2015 and concluding on 21st November 2017. In both cohorts, accelerometer data were collected throughout the entire year, independent of the season, ensuring a comprehensive dataset across different times of the year. The methodological quality of the studies were assessed using the Strengthening the Reporting of Observational Studies in Epidemiology (STROBE) for a cross-sectional study [25].

### Ethics statement

The Cuenca study protocol was approved by the Ethics Committee of Universidad San Francisco de Quito (No. 2017-090E). The Quinindé study protocol was approved by the Ethics Committee of Hospital Pedro Vicente Maldonado (2005; for cohort follow-up to 5 years) and Universidad San Francisco de Quito (2010; for cohort follow-up to 8 years). In both studies, parents or guardians provided written informed consent, and assent was obtained from the children. In the case of the ECUAVIDA cohort, the children’s assent was requested once the participants reached the age of 8 years.

### Study procedures–data collection

Data on PA intensity, anthropometric measurements, and socialdemographics variables were collected using similar procedures across both study samples to address potential sources of bias.

#### Physical activity intensity assessment

**Fig 1:**
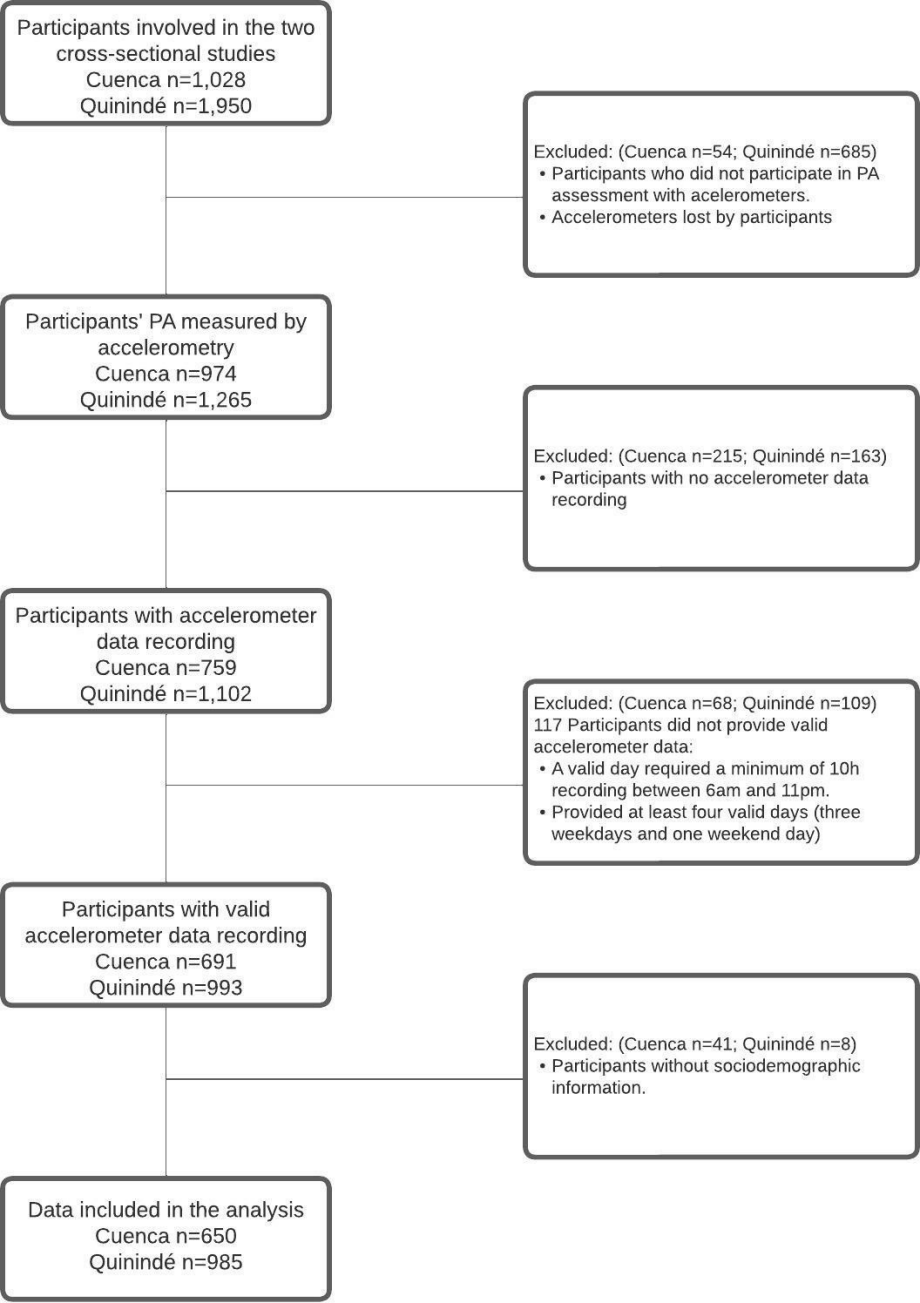
Flowchart of participant accelerometry data exclusion.

Time spent in SB and PA at different intensities were objectively measured using ActiGraph GT1M and GT3X+ accelerometers (ActiGraph, Fort Walton Beach, FL, USA), which have demonstrated high reliability for use in children, particularly in higher altitude settings [26, 27]. In Cuenca, the distribution of the accelerometers was managed by the research team in coordination with the educational centers, occurring one hour before the scheduled start time and collection one hour after the scheduled device end time. In the ECUAVIDA cohort, the use of accelerometers was explained in person (in the presence of the child’s legal representative) to each participant at the cohort clinic in Quininde immediately prior to the scheduled start time and collection was done in person at the cohort clinic at the time of the scheduled finish time. Participants were asked to wear the accelerometers in an elastic belt on the right hip during waking hours for 7 consecutive days. Acelerometers could only be removed during water-related activities and at night before sleep. The initialization parameters, such as the data collection period (bouts) and the number of collection axes (mono-, di-, tri-axial), were defined as described elsewhere [28]. A valid day required a minimum of 10h recording between 6am and 11pm, and non-wear time was defined as 60 min of consecutive zero counts with an allowance for 2 min of interruptions [29]. Children who provided at least four valid days (three weekdays and one weekend day) were included in the analyses (Fig 1) [28, 30]. Sedentary, light, moderate, vigorous, and moderate-vigorous physical activity were expressed as counts per minute and classified using Evenson’s cut-points which have been reported to provide greater accuracy for children and adolescents [31]. Sedentary, light, moderate, vigorous PA were defined as 0–100, 101–2295, 2296–4011, and ≥4012 counts/minute, respectively [32]. Children with a daily average of at least 60 minutes of moderate-vigorous physical activity (MVPA) were categorized as meeting the WHO PA recommendations [2]. Initialization of accelerometers, data download, and conversion of count to minutes was done using ActiLife (version 6.0, ActiGraph Corp, Pensacola, FL, USA) using standardized procedures. PA time sampling interval (epoch) was set to 15 s.

#### Anthropometric measurements

Body weight and height were measured by trained personnel using standardized techniques [33]. Weight was recorded to the nearest 0.10 kg using digital scales (SECA 803. Hamburg. Germany), and height was registered to the nearest mm using portable stadiometers (SECA 213 Germany). Waist circumference (WC) was recorded at the minimum circumference in centimeters (cm) between the iliac crest and rib cage using an anthropometric tape (LUFKIN 2m W606PM). Anthropometric measurements were done in schools in Cuenca and in a dedicated cohort clinic in Quininde. All measurements were performed in the presence of a teacher or legal representative. Fat mass percentage was obtained by a prediction model of fat mass validated in children and adolescents described in detail elsewhere [34]. Weight, height, and waist circumference were recorded in duplicate, considering a third only in the case of a difference exceeding 2%. Body mass index (BMI) was calculated as the child’s kg/m² and used to categorize children into 4 groups by nutritional status (malnutrition, normal weight, overweight, and obesity), defined using WHO standards based on the age and sex-specific BMI Z-scores [35]. Abdominal obesity was considered when the waist circumference percentile was equal to or greater than the 90th percentile according to age and sex [36, 37].

#### Socialdemographic determinants

**Fig 2:**
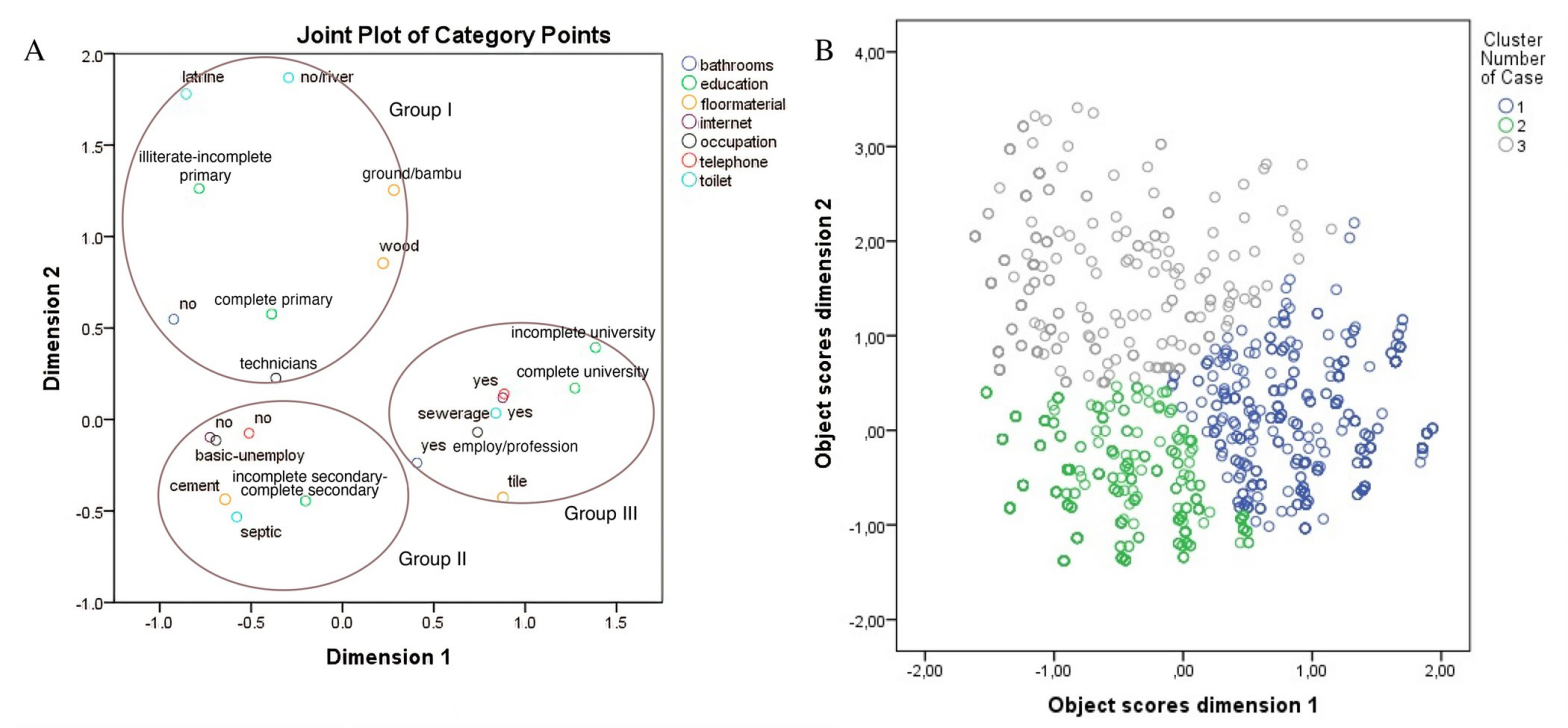
Multiple correspondence analysis (MCA). MCA was performed using seven sociodemographic factors, including having a bathroom (Yes/No), father’s/mother’s education (illiterate or completed primary education; incomplete or completed secondary education; and incomplete or completed higher education), type of flooring in the home (soil, bamboo, cement, tile, or wood), access to the internet (Yes/No), father’s/mother’s occupation (basic or unemployed; technician or craftsman; employee or professional), having a home telephone (Yes/No), and type of sanitation system (None/river, latrine, septic tank, or sewage). A) Variables natural tendency groups by socioeconomic class (low, middle, and high) using the first two dimensions of MCA. Group 1 (low): Households without a sanitation system or those using river/latrine-based sanitation; no bathroom is available. Parental education is either illiterate or limited to incomplete or primary education. Building materials include ground, bamboo, or wood. Occupations are predominantly technical. Group 2 (middle): Households lacking internet access and a home telephone. Parental education ranges from incomplete to complete secondary education. Occupations are basic or parents are unemployed. Flooring material is cement, and the sanitation system is a septic tank. Group 3 (high): Households with both a home telephone and internet access. These homes have a bathroom with a sewage-based sanitation system. Parental occupations are employed or professional, with incomplete or complete university education. Flooring material is ceramic. B) Cluster plot showing MCA object scores assigned to each individual (points). The colors represent socioeconomic groups (gray–low; green -medium; brown–high).

A validated questionnaire assessing children and parents’ sociodemographic factors was completed by parents/guardians. Socialdemographic variables included age, ethnicity, sex, type of sanitation system, access to the internet, having a home telephone landline, father’s/mother’s education, father’s/mother’s occupation, and type of flooring in the home. Ethnicity in Cuenca was based on the child’s self-report, while in the Quinindé, data were provided by parents. Ethnicity was categorized into Mestizo (the offspring of Indian and white parents) and non-Mestizo due to the fact that 77.4% of the Ecuadorian population defines itself as Mestizo [38]. Indigenous, Afro-descendants, Montubio, and White groups were combined as a single non-Mestizo grouping. Father’s/mother’s education was classified into 3 levels: illiterate or complete primary education, incomplete or complete secondary education, and incomplete or complete higher education. Father’s/mother’s occupation included a) basic or unemployed (non-active or unemployed workers), b) technician and craftsman (plant and machine operators, journeymen, craftsmen, skilled agricultural and fishing workers), and c) employee or professional (service and commercial workers, office employees, technicians, mid-level professionals, armed forces, scientific and intellectual professionals, and management personnel in public administration and companies). The highest level of education or occupation of the father was selected if it was equal to or higher than the mother’s level of education or occupation and vice versa. The floor type of the house was classified into two categories: 1) soil/bamboo/cement and 2) ceramic/wood. Sociodemographic variables (father’s/mother’s education; father’s/mother’s occupation; access to sanitation, internet, and home phone landline; material goods in the household and type of sanitation system) were used to define socioeconomic status (SES) into three categories (low, medium, and high) using multiple correspondence analysis (MCA) (Fig 2). MCA is a statistical technique commonly used in social sciences to analyze and visualize relationships between categorical variables [39]. Participants were allocated to their respective SES groups and defined a categorical variable subsequently used as an explanatory variable in the analyses.

### Statistical analysis

Data from Cuenca and Quinindé are presented separately due to differences between the populations that the samples represent. Sociodemographic and household factors of participants were summarized using frequencies (percentages). Descriptive statistics for PA intensity metrics were presented in terms of measures of central tendency (means, standard deviations, interquartile) for continuous variables and proportions for categorical ones. A Spearman correlation matrix was performed as an exploratory analysis to provide a comprehensive overview of the relationships among different PA intensity metrics. Sedentary time, light, moderate, and vigorous PA, measured in minutes per day as described above, were log-transformed, and considered components of a multivariate normally distributed response variable analyzed using multivariate regression techniques. Estimates for associations between the hypothesized explanatory variables (sex, ethnicity, WC, and SES) and PA intensity outcomes were back-transformed as geometric mean ratios (GMR) and interpreted as fold-changes in the response component for one unit (or an epidemiologically relevant unit) increase in an explanatory variable. Such a multivariate approach is preferable to a series of univariate regressions, as their correlations are intrinsically considered. We used the Breusch–Pagan test to assess the presence of heteroskedasticity in the model with a p-value less than 0.05, rejecting the null hypothesis of presence of residuals’ heteroscedasticity. MVPA was also presented as a binary outcome to indicate whether a child met the WHO PA guidelines of at least 60 minutes of MVPA daily or not. Logistic regression was used to investigate the relationship between this outcome and relevant independent variables (sex, ethnicity, WC, and SES), with univariate analyses and a multivariable model incorporating all such variables. To manage missing data, only cases with complete data for all relevant variables in the multivariate regressions were included. Adequate statistical power was maintained in the analyzed data set to identify any associations. Age was only presented as a summary statistic, as this variable had minimal variability within the two samples. WC was selected among the anthropometric factors because of its better predictive value for future cardiometabolic risk in children and adolescents, compared to weight and height [40]. Associations between WC and the variables of interest were considered for every 5 cm increase in WC. All analyses were done using Stata 17 (StataCorp. 2021. *Stata Statistical Software*: *Release 17*. College Station, TX: StataCorp LLC.), and statistical significance was inferred by p<0.05.

## Results

### Characteristics of study samples

Table 1 shows data for sociodemographics, household factors, anthropometrics, weight status, and descriptive summaries for each PA metric in the two samples of children from Cuenca and Quinindé. There were marked differences between children in samples from Cuenca and Quinindé; the mean age of the Cuenca sample was greater (10.8 vs. 8.3 years in Quinindé); SES of the Cuenca sample was higher with 79.3% of households being categorized as high SES compared with 10.9% in Quinindé; and Cuenca households were much more likely to have a sewage connection, have internet, a telephone landline, and ceramic or wood flooring. Because of these systematic differences, the two samples were analyzed separately. Regarding weight status, children in Cuenca were more likely to be overweight or obese than those in Quinindé (35.5% vs. 19.9%), although proportions with abdominal obesity based on waist circumference were similar (Cuenca 10.3% vs. Quinindé 10.1%). The mean MVPA minutes/day for both Cuenca (62.1 ± 39.2) and Quinindé (77.9 ± 28.8) children were slightly above the recommended 60 min/day. Proportions of children meeting daily WHO recommendations were greater in Quinindé than in Cuenca (38.9% vs. 70.0%).

**Table 1:** Distributions or frequencies of variables for sociodemographics, household factors, anthropometrics, and nutritional status, and physical activity metric in 1,635 children from Cuenca and Quininde, Ecuador.

*Variables*	*Summary /Categories*	*Cuenca (N = 650)*	*Quinindé (N = 985)*
**SOCIODEMOGRAPHICS**			
**Age (years)**	Mean [SD]	10.83 [1.12]	8.32 [0.41]
	Median [Q1; Q3]	10.87 [9.89; 11.8]	8.22 [8.07; 8.42]
**Sex**	Girls (%)	343 (52.77%)	477 (48.43%)
	Boys (%)	307 (47.23%)	508 (51.57%)
**Ethnicity**	Mestizo (%)	503 (77.38%)	712 (72.28%)
	Other (%)	147 (22.62%)	273 (27.72%)
**Educational levels of household head**	Illiterate/primary Education (%)	136 (22.55%)	264 (29.63%)
	Secondary Education (%)	249 (41.29%)	567 (63.64%)
	Advanced Education (%)	218 (36.15%)	60 (6.73%)
**Occupation of household head**	Basic/Unemployed (%)	73 (12.92%)	437 (49.05%)
	Technician/Craftsman (%)	110 (19.47%)	235 (26.37%)
	Employee/Professional (%)	382 (67.61%)	219 (24.58%)
**Socioeconomic Status**	Low (%)	59 (9.62%)	196 (22.00%)
	Medium (%)	68 (11.09%)	598 (67.12%)
	High (%)	486 (79.28%)	97 (10.89%)
**HOUSEHOLD FACTORS**			
**Sanitation system**	No (%)	95 (15.81%)	777 (87.21%)
	Yes (%)	506 (84.19%)	114 (12.79%)
**Internet**	No (%)	127 (21.03%)	691 (77.55%)
	Yes (%)	477 (78.97%)	200 (22.45%)
**Conventional phone**	No (%)	248 (40.86%)	704 (79.01%)
	Yes (%)	359 (59.14%)	187 (20.99%)
**Flooring**	Soil/Bamboo/Cement (%)	112 (18.54%)	589 (66.11%)
	Ceramics/Wood (%)	492 (81.46%)	302 (33.89%)
**ANTHROPOMETRICS AND NUTRITIONAL STATUS**			
**Weight (kg)**	Mean [SD]	37.34 [9.87]	25.99 [5.35]
	Median [Q1; Q3]	35.85 [29.80; 42.60]	24.90 [22.55; 28.00]
**Height (cm)**	Mean [SD]	139.48 [9.25]	125.48 [5.74]
	Median [Q1; Q3]	139.08 [132.40; 145.35]	125.40 [121.65; 129.05]
**Waist circumference (cm)**	Mean [SD]	66.23 [8.61]	58.14 [6.41]
	Median [Q1; Q3]	64.90 [59.75; 71.25]	56.75 [54.10; 60.40]
**Fat mass (kg)**	Mean [SD]	10.71 [5.27]	6.49 [2.94]
	Median [Q1; Q3]	9.58 [6.69; 13.23]	5.79 [4.63; 7.23]
**BMI Z-score**	Mean [SD]	0.56 [1.20]	0.12 [1.17]
	Median [Q1; Q3]	0.57 [-0.32; 1.43]	0.03 [-0.64; 0.74]
**Nutritional status**	Normal (%)	412 (63.38%)	768 (77.97%)
	Underweight (%)	7 (1.08%)	21 (2.13%)
	Overweight (%)	155 (23.85%)	121 (12.28%)
	Obese (%)	76 (11.69%)	75 (7.61%)
**Abdominal obesity**	No (%)	583 (89.69%)	886 (89.95%)
	Yes (%)	67 (10.31%)	99 (10.05%)
**PHYSICAL ACTIVITY INTENSITY METRICS**			
**Sedentary time (min/day)**	Mean [SD]	545.28 [113.08]	479.95 [96.56]
	Median [Q1; Q3]	526.05 [464.94; 612.54]	484.19 [414.33; 553.38]
**Light PA (min/day)**	Mean [SD]	305.24 [64.60]	370.04 [53.22]
	Median [Q1; Q3]	308.39 [269.71; 348.16]	370.71 [338.17; 402.71]
**Moderate PA (min/day)**	Mean [SD]	47.28 [35.33]	57.83 [19.50]
	Median [Q1; Q3]	39.73 [29.75; 51.73]	55.98 [43.14; 70.38]
**Vigorous PA (min/day)**	Mean [SD]	14.75 [8.78]	20.06 [11.21]
	Median [Q1; Q3]	12.93 [8.25; 18.96]	17.88 [11.79; 26.02]
**Moderate-Vigorous PA (min/day)**	Mean [SD]	62.07 [39.22]	77.89 [28.77]
	Median [Q1; Q3]	53.09 [39.83; 72.17]	75.08 [56.42; 96.13]
**Meet WHO PA guidelines**	No (%)	397 (61.08%)	296 (30.05%)
	Yes (%)	253 (38.92%)	689 (69.95%)

Missing data Cuenca: educational level of household head (n = 47), occupation of head of household (85), socioeconomic status (37), sanitation system (49), home internet (46), home landline (43), and flooring (46). Missing data Quinindé: for same variables (n = 94).

### Determinants of minutes of physical activity intensity

Table 2 shows correlations between different PA intensity metrics for children in Cuenca and Quinindé. There was some evidence of correlations between the different metrics (analyzed as continuous variables), being generally negative between sedentary time and other metrics and positive between metrics of non-sedentary activity. Associations with relevant sociodemographic and other variables in univariate and multivariable analyses are shown in Table 3 separately for the two samples. All explanatory variables were included in multivariate models, as each one proved significant for at least one PA metric.

**Table 2:** Spearman correlation matrix of physical activity intensity by city.

	Sedentary time	Light PA	Moderate PA	Vigorous PA
**Cuenca**				
Sedentary time [min/day]	1			
Light PA [min/day]	**-0.5802**	1		
Moderate PA [min/day]	0.0699	**-0.309**	1	
Vigorous PA [min/day]	**-0.3101**	**0.2386**	**0.3197**	1
**Quinindé**				
Sedentary time [min/day]	1			
Light PA [min/day]	**-0.1746**	1		
Moderate PA [min/day]	**-0.356**	**0.398**	1	
Vigorous PA [min/day]	**-0.1943**	**0.0898** **	**0.7358**	1

Significant r values are given in bold. Two asterisks (**) denote significance at P < 0.01.

**Table 3:** Univariate and multivariable associations between anthropometric and sociodemographic variables and physical activity intensity metrics derived from multivariate regression analyses.

	*Sedentary time*				*Light PA*				*Moderate PA*				*Vigorous PA*			
	*Univariate*		*Multivariable*		*Univariate*		*Multivariable*		*Univariate*		*Multivariable*		*Univariate*		*Multivariable*	
Variable	GMR [95% CI]	*P value*	Adjusted GMR [95% CI]	*P value*	GMR [95% CI]	*P value*	Adjusted GMR [95% CI]	*P value*	GMR [95% CI]	*P value*	Adjusted GMR [95% CI]	*P value*	GMR [95% CI]	*P value*	Adjusted GMR [95% CI]	*P value*
**CUENCA**																
Sex (Boys vs. Girls)	0.9719 [0.9418; 1.0030]	0.076	0.9708 [0.9406; 1.0019]	0.065	1.0173 [0.9780; 1.0582]	0.393	1.0242 [0.9845; 1.0656]	0.235	1.3011 [1.2075; 1.4019]	< 0.001	1.2815 [1.1897; 1.3804]	<0.001	1.4199 [1.3000; 1.5508]	< 0.001	1.3922 [1.2729; 1.5226]	<0.001
Ethnicity (Non-mestizo vs. Mestizo)	0.9777 [0.9416; 1.0152]	0.241	0.9956 [0.9587;1.0339]	0.819	1.003 [0.9571; 1.0515]	0.895	0.9930 [09470; 1.0412]	0.771	1.1592 [1.0577; 1.2704]	0.002	1.1075 [1.0131; 1.2106]	0.025	1.1776 [1.0556; 1.3136]	0.003	1.0854[0.9749; 1.2083]	0.134
Waist circumference (per 5cm)^a^	1.0049 [1.0031; 1.0067]	< 0.001	1.0048 [1.0029; 1.0066]	<0.001	0.9959 [0.9936; 0.9982]	< 0.001	0.9953 [0.9930; 0.9977]	<0.001	0.9948 [0.9904; 0.9993]	0.023	0.9979 [0.9936; 1.0023]	0.345	0.9860 [0.9808; 0.9911]	< 0.001	0.9876 [0.9824; 0.9928]	<0.001
SES																
Medium vs. low	0.9872 [0.9160; 1.0543]	0.627	0.9856 [0.9201; 1.0559]	0.680	1.0745 [0.9846; 1.1727]	0.107	1.0711 [0.9824; 1.1679]	0.119	0.9779 [0.8261; 1.1575]	0.794	0.9698 [0.8245; 1.1406]	0.711	1.0279 [0.8348; 1.2656]	0.795	1.0105 [0.8310; 1.2288]	0.917
High vs. low	1.1085 [1.0497; 1.1706]	< 0.001	1.0982 [1.0409; 1.1587]	0.001	1.0042 [0.9385; 1.0746]	0.903	1.0133 [0.9473; 1.0838]	0.701	0.7727 [0.6780; 0.8805]	< 0.001	0.7652 [0.6744; 0.8682]	<0.001	0.8435 [0.7179; 0.9911]	0.039	0.8499 [0.7299; 0.9897]	0.036
QUININDÉ																
Sex (Boys vs. Girls)	0.9731 [0.9472; 0.9998]	0.048	0.9690 [0.9419; 0.9970]	0.030	1.0053 [0.9868; 1.0242]	0.573	1.0015 [0.9821; 1.0212]	0.882	1.3420 [1.2881; 1.3982]	< 0.001	1.3513 [1.2955; 1.4095]	< 0.001	1.5698 [1.4640; 1.6832]	< 0.001	1.5798 [1.4724; 1.6950]	< 0.001
Ethnicity (Non-mestizo vs. Mestizo)	1.0204 [0.9900; 1.0517]	0.191	1.0220 [0.9903; 1.0548]	0.176	1.0018 [0.9812; 1.0228]	0.863	1.0004 [0.9790; 1.0224]	0.969	1.0765 [1.0240; 1.1317]	0.004	1.0838 [1.0342; 1.1357]	0.001	1.1506 [1.0583; 1.2509]	0.001	1.1617 [1.0744; 1.2562]	< 0.001
Waist circumference (per 5cm)	0.9993 [0.9972; 1.0014]	0.512	0.9992 [0.9969; 1.0015]	0.497	0.9991 [0.9976; 1.0005]	0.204	0.9986 [0.9970; 1.0002]	0.085	0.9976 [0.9941; 1.0011]	0.173	0.9980 [0.9945; 1.0014]	0.247	0.9876 [0.9819; 0.9934]	< 0.001	0.9893 [0.9836; 0.9950]	< 0.001
SES																
Medium vs. low	1.0348 [0.9993; 1.0716]	0.055	1.0376 [1.0018; 1.0747]	0.040	0.9927 [0.9692; 1.0167]	0.546	0.9952 [0.9714; 1.0195]	0.694	0.9161 [0.8651; 0.9701]	0.003	0.9056 [0.8596; 0.9541]	< 0.001	0.9482 [0.8624; 1.0424]	0.271	0.9447 [0.8659; 1.0306]	0.199
High vs. low	1.0983 [1.0419; 1.1577]	< 0.001	1.1009 [1.0441; 1.1607]	< 0.001	0.9906 [0.9555; 1.0271]	0.609	0.9943 [0.9588; 1.0311]	0.757	0.8414 [0.7717; 0.9174]	< 0.001	0.8376 [0.7743; 0.9061]	< 0.001	0.8830 [0.7654; 1.0188]	0.088	0.8944 [0.7845; 1.0197]	0.095

The GMR represent the fold changes associated with one unit (or relevant quantity) increase in a continuous variable or with a change in the level of a categorical variable relatively to the baseline.

Boys spent more time doing MPA and VPA than girls in both settings: in Cuenca, time spent by boys doing MPA and VPA was 28% (95% CI 19%- 38%) and 39% (95% CI 27%- 52%) greater than for girls, respectively; while in Quinindé the equivalent times spent by boys compared with girls were 35% (95% CI 30%- 41%) and 58% (95% CI 47%- 69%) greater. The adjusted analysis in the Quinindé sample also showed that boys spent significantly less time sedentary than girls (GMR 0.97, 95% CI 0.94, 0.99). Non-Mestizo children were predominantly Afro-Ecuadorian in Quinindé and predominantly White in Cuenca. In adjusted analyses, non-Mestizo children spent more time than Mestizo children in MPA in both settings (by 11%, 95% CI 1–21% in Cuenca and by 8%, 95% CI 3–14% in Quinindé), and longer time in VPA in Quinindé (by 16%, 95% CI 7–26%). Waist circumference was positively associated with sedentary time and inversely with light PA (LPA) and VPA in Cuenca; the effect sizes were small albeit statistically significant. In Quinindé, WC was also inversely associated with vigorous PA. Children from economically advantaged spent 10% (95% CI 4–16%), longer sedentary periods compared to their poorer counterparts in both Cuenca and Quinindé. Similarly, these more affluent children spent 23% (95% CI 13–33%) and 15% (95% CI 1–27%) less time in MPA an VPA in Cuenca, respectively. In Quinindé the more affluent children spent (16%, 95% CI 9–23%) less time in MPA compared to the least affluent.

### Determinants of meeting WHO recommendations for physical activity

Table 4 shows univariate and multivariable associations between potential risk factors and attainment of the WHO recommendations for MVPA in the Cuenca and Quinindé samples. In multivariable analyses, boys were around three times more likely than girls (adj. OR 3.09, 95% CI 2.17–4.39) to meet MVPA in Cuenca and almost six times more likely to meet MVPA in Quinindé (adj. OR 5.63, 95% CI 4.03–7.85). Non-Mestizos in Quinindé but not Cuenca were significantly more likely than Mestizos to meet MVPA (adj. OR 1.45, 95% CI 1.02–2.07). Waist circumference was significantly inversely associated with meeting MVPA in Cuenca (per 5cm increase, adj. OR 0.96, 95% CI 0.94–0.98). The most affluent children were much less likely to attain daily MVPA compliance compared to the poorest in both settings (Cuenca, adj. OR 0.45, 95% CI 0.25–0.81; Quinindé, adj. OR 0.55, 95% CI 0.31–0.97).

**Table 4:** Attainment of WHO recommendations for moderate-vigorous physical activity (MVPA) and associations with anthropometric and sociodemographic variables in children from Cuenca and Quinindé.

	Cuenca [n = 650]		Quininde [n = 985]	
Variables	Meeting MVPA [N/Y]		Meeting MVPA [N/Y]	
	Univariate	Multivariable	Univariate	Multivariable
	OR [95% CI] P Value	Adj. OR [95% CI] P Value	OR [95% CI] P Value	Adj. OR [95% CI] P Value
Sex (Boys vs. Girls)	**3.09 [2.23; 4.30] < 0.001**	**3.09 [2.17; 4.39] < 0.001**	**5.11[3.76; 6.93] < 0.001**	**5.62 [4.03; 7.85] < 0.001**
Ethnicity (Non-Mestizo vs. Mestizo)	**1.65 [1.14; 2.39] 0.008**	1.32 [0.87; 1.99] 0.191	**1.38[1.01; 1.90] 0.043**	**1.45 [1.02; 2.07] 0.039**
Waist circumference (5 cm effect)	**0.95 [0.93; 0.97] < 0.001**	**0.96 [0.94; 0.98] < 0.001**	0.98[0.96; 1.00] 0.074	0.98 [0.95; 1.00] 0.062
SES				
Medium vs. low	1.02 [0.51; 2.04] 0.964	0.95 [0.45; 1.99] 0.894	0.81[0.56; 1.16] 0.250	0.75 [0.50; 1.11] 0.145
High vs. low	**0.48 [0.28; 0.83] 0.008**	**0.45 [0.25; 0.81] 0.007**	**0.59[0.35; 1.00] 0.049**	**0.55 [0.31; 0.97] 0.040**

## Discussion

We present the first objective assessment of PA intensity in children aged 8–12 years from Ecuador. This study investigated two different populations of school-age children living in highland urban (Cuenca) and tropical rural (Quinindé) settings. Our results revealed variations in mean minutes of MVPA per day, prevalence, and factors influencing adherence to WHO daily PA recommendations, with higher levels generally observed in rural settings.

The average daily time spent by children in Cuenca on MVPA aligns with results reported in a multinational and cross-sectional study that included representative data from children aged 9–11 years analyzed in 12 countries with highly variable levels of affluence [41]. Nevertheless, it is less than the average reported by our participants from the rural context, where an additional 15 minutes per day is observed compared to the urban setting. Similar trends of increased daily MVPA and higher prevalence of compliance with these recommendations in rural children and lower in urban participants were observed in investigations conducted in Kenya and Mozambique [13, 14]. In fact, participants from rural settings spend an average of 75 minutes per day on MVPA, which may be attributed to living in rural settings where children exhibit higher levels of outdoor activities or active transportation [42]. In addition, children and adolescents actively traveling to school accumulated around 10–14 more minutes of MVPA than passive car travelers and are more likely to be active in other contexts [43, 44]. However, another possible explanation for these differences in urban and rural settings could be the necessity- versus choice-based physical activity models framework [45]. Quininde, in contrast to Cuenca, exhibits higher levels of poverty and limited access to essential services, including sanitation, healthcare, and public transportation, forcing rural children to walk to school by necessity rather than as a healthy lifestyle choice. More research is needed to identify participation in need-based versus choice-based physical activity in different PA domains in urban and rural areas in Latin America.

The existing body of literature has consistently reported differences in PA levels between sexes, with a lower quantity of MVPA minutes and a lower prevalence of adherence to WHO PA recommendations among girls as compared to boys [14, 18, 46]. Our study corroborates the sex disparity in MPA, VPA, and attainment of WHO PA recommendations across urban and rural environments. Lower participation in organized sports and parents’ safety concerns have been described as some mechanisms underlying this difference [47]. Due to higher safety concerns, parents are more protective of their daughters than their sons, limiting girls’ active commuting and PA [48]. Previous data from Cuenca and from a close rural area has suggested that traffic and crime in urban areas and agriculture and regulations in rural areas are barriers for girls engaging in PA in their leisure time [49]. Moreover, in Quinindé, an expected gender social-specific role is observed. Girls tend to perform care and support tasks for older women in the household before and after school, decreasing the possibility of being more active in their leisure time [50]. Our findings suggested that girls from urban and rural contexts engaged less in MPA an VPA and were less likely to comply WHO PA recommendations. Interventions designed to promote PA, with a specific emphasis on gender considerations, are imperative to increase MVPA levels in girls in their leisure-time PA and improve adherence to WHO guidelines on PA.

Findings regarding associations between ethnicity and PA metrics are inconsistent among studies. Some studies in children revealed correlations between these two variables, while others reported a lack of association [51–53]. Our results suggest that, in both Cuenca and Quinindé, non-Mestizo children were more physically active than their counterparts; however, only in Quinindé non-Mestizos (primary Afro-Ecuadorian) were more likely to meet daily MVPA recommendations when compared with Mestizos. In concordance with our results, the *USA National Health and Nutrition Examination Survey*, *2007–2016*, reported that White and Black adolescents spend more time on MVPA than their Hispanic peers (proxy of Mestizo) [54], specifically Black children are more active based on activity counts and time spent in MPA and VPA [51] as observed in our Non-Mestizo participants from Quinindé. No evidence has been reported on how ethnicity may influence preference for or participation in higher intensity PA among children in Ecuador. However in rural setting, open spaces provide more opportunities to engage in vigorous sports such as soccer or basketball, unlike urban cities that offer a wide diversity of physical activities [42, 55]. These disparities highlight the importance of designing interventions explicitly tailored to each ethnic group to enhance overall physical activity in children.

Higher WC was associated with more sedentary time and less time in LPA, MPA, and VPA only from Cuenca. Our findings align with a longitudinal study suggesting that adiposity impacted SB and influenced children’s physical activity levels [56, 57]. In addition, a separate longitudinal study involving children aged 8 to 11 years revealed that higher levels of fatness predicted a reduction in physical activity and an increase in sedentary time [58]. This directional association between adiposity and SB and PA in children could be explained by exercise psychology. Children with higher WC may experience body image concerns about their appearance and abilities, which could discourage them from participating in PA and lead to a more sedentary lifestyle and less likelihood to accumulate 60 minutes/day of MVPA [59–61]. Moreover, dissatisfaction with body weight intensifies across adolescents and may have a negative impact on healthy behavior [62]. Research aimed at addressing the psychological factors underlying the increase in anthropometric metrics is needed to address body image concerns and unfavorable body compositions, as well as to combat SB and promote higher levels of PA among urban children in Ecuador.

Healthy behaviors, such as being physically active, are not only influenced by individual knowledge and attitudes but also depend on the environment in which children live, play, and learn in their community [63]. In addition, the built environment plays a significant role in promoting children’s PA [64]. Urban sprawl, unsightly views, poorly maintained roads and facilities, a dirty environment, litter, or broken glass, have all been shown to have negative effects on PA [65]. Children without access to parks were more likely to be inactive every week and to spend four hours or more in daily screen time [66]. Furthermore, girls living in neighborhoods with two or three traffic lights were more likely to walk/cycle trips than those living in neighborhoods with fewer traffic lights, while for boys, living on a dead-end street facing a through street was associated with an increase of 9, 5 and 22 minutes in PA performed after school, in the evenings and on weekends, respectively [67]. In contrast, availability and accessibility in proximity to green spaces, parks, recreational facilities, and sidewalks were found to be associated with increased PA levels, park-based PA, reduced SB, and/or active commuting among all age groups [68, 69]. Building favorable physical environments, through initiatives such as building new exercise facilities and improving the accessibility of existing ones, can promote healthy behaviors and health benefits [20].

Our findings suggested that affluent children from both cohorts are more sedentary and less likely to achieve daily MVPA guidelines. Similar associations between SES and SB have been described in LMIC; the average time devoted to computers, video games, and non-screen-related sedentary leisure has been reported as greater in high SES than in low SES groups [70, 71]. One possible explanation is that families with higher household incomes have greater access to purchasing electronic devices, displacing time for active behavior [72, 73]. In contrast to our results, evidence indicates that SES correlates positively with PA [74]. Children from higher SES tend to participate more in organized sports outdoors than children from low-SES families, thereby contributing to meeting the recommended levels of MVPA [75]. The findings suggest that public health policy should be oriented to identify factors contributing to SB and PA behavior in children from affluent socioeconomic backgrounds in upper-middle countries.

There are limited published accelerometer-derived data on SB and PA levels and their sociodemographic and anthropometric determinants from populations of children living in upper-middle-income countries such as Ecuador. This study has several strengths, including using representative samples of participants from urban and rural settings and using objective measures to evaluate PA. An additional strength is the mitigation of potential underestimation of PA intensity levels by restricting the analysis of accelerometer data to waking hours. Limitations include the lack of questionnaires to assess the minutes spent on screen time and the specific types PA—whether light, moderate, or vigorous—in which participants engaged. Additionally, variations in the timing of studies, participant ages, and methods of collecting ethnic data—such as relying on data provided by parents in Quinindé—limited our ability to make quantitative comparisons between samples. Finally, the cross-sectional design of the data restricts the ability to draw causal conclusions.

## Conclusion

Sex, ethnicity, waist circumference, and SES disparities were associated with variations in PA intensity levels among Ecuadorian schoolchildren from urban and rural populations. Intervention strategies to enhance healthy and active lifestyles in children should consider anthropometric and sociodemographic perspectives. An understanding of the usefulness of measurements such as waist circumference will be important for the design of personalized PA programs that address individual body composition and fat distribution. Furthermore, considering sociodemographic factors aids in identifying specific barriers and facilitators to PA among different subgroups. Profiling these factors enables the development of targeted and culturally relevant programs that address the unique needs of diverse populations.

## Supporting information

S1 Dataset (XLSX)

S1 Checklist STROBE statement—checklist of items that should be included in reports of *cross-sectional studies*. (DOCX)
